# Avoiding Cutaneous Melanoma Requires Proper Sun Protection and a Correspondingly High Level of Education: A Case Control Study

**DOI:** 10.1111/jocd.16677

**Published:** 2024-12-10

**Authors:** Zeynep Altan Ferhatoglu, Faruk Tas

**Affiliations:** ^1^ Dermatology and Venereology Department, Cerrahpaşa Medical Faculty Istanbul University‐Cerrahpaşa Istanbul Turkey; ^2^ Medical Oncology Department Institute of Oncology, Istanbul University Istanbul Turkey

**Keywords:** behavior and education, melanoma, sun protection

## Abstract

**Background/Aim:**

Cutaneous melanoma ranks 5th among all cancers in terms of estimated new case rates in men and women (6% and 4%, respectively). The most consistent known modifiable risk factor for a cancer of this severity is exposure to ultraviolet rays. In this study, we aimed to compare the sun protection knowledge, habits and behaviors of CM patients with healthy volunteers. In addition, we aimed to determine the level of change in sun protection attitude of CM patients before and after diagnosis.

**Materials and Methods:**

In this study, the knowledge levels and habits of cutaneous melanoma patients regarding sun protection were questioned through surveys and compared with the control group. The surveys were developed taking into consideration the Turkish Dermatology Association's sun protection patient information recommendations.

**Results:**

The Male:Female (M: F) ratio of 86 melanoma patients was 1.52, and the median age was 51 ± 15 years. In the control group of 174 participants, the M:F ratio was 1.38 and the median age was 49 ± 10 years. The education level of melanoma patients was statistically significantly lower than the control group (*p* < 0.001). Compared to melanoma patients, participants in the control group had more accurate information on 5 questions about sunscreen application methods (applying at least 30 min before sun exposure, renewing after 2–4 h in sunny environments, renewing after the sea/pool, renewing after sports, amount of application) (*p* < 0.001 for each of the 5 questions).

**Conclusion:**

The results of our study revealed that the education level in CM patients was statistically significantly lower than in the control group of similar age and gender. In addition, in the control group, parallel to the education level, the level of sun protection knowledge was higher, and sun avoidance/protection behaviors were closer to what they should be.

## 1 Introduction

Cutaneous melanoma ranks 5th among all cancers in terms of estimated new case rates in men and women (6% and 4%, respectively) [1]. Despite that there is disagreement as to whether this is an increase in the true occurrence of disease or in the diagnosis, it has been determined that CM, which was once rare, has increased 6‐fold compared to 40 years ago [2]. The risk of cutaneous melanoma generally increases with age and is more common in older populations. On the other hand, cutaneous melanoma is reported to be among the most common cancers in young adults [3]. Melanoma formation is the result of a multifactorial developmental process. It requires exposure to certain environmental factors for sufficient periods of time on the basis of genetic predisposition. Established risk factors of CM include ultraviolet (UV) radiation exposure, fair skin, male gender, and older age [4].

The strongest phenotypic feature reported for CM in the literature is a high number of acquired nevi (e.g., more than 20 nevi larger than 5 mm in diameter; criteria can vary in different studies). The risk increases 5–10 times in individuals with a high number of nevi, and in addition, this situation appears to be related to the early age of diagnosis. It is also known that excessive exposure to UV radiation increases the number and diameter of acquired nevi [5].

Over 75% of CMs in White populations are considered to develop due to the mutagenic effect of UV radiation [6]. Ultraviolet radiation is the most consistently reported modifiable risk factor for CM. Ultraviolet‐B radiation (wavelength: 280–320 nm) contributes to CM formation through direct deoxyribonucleic acid (DNA) damage and UV‐A radiation (wavelength: 320–400 nm) through indirect DNA damage by causing oxidative stress [7].

Ultraviolet radiation exposure leading to sunburn has been shown to increase the risk of melanoma by 2.03 times. (RR = 2.03; 95% CI: 1.73, 2.37) [8] In order for a disease to increase in prevalence in society beyond the impact of a defined risk factor, there must also be an increase in the frequency of exposure to the risk factor in that society. In a recent article, the authors emphasize that such a situation develops for CM [2]. In addition, the risk of developing melanoma again is 9 times higher in melanoma survivors compared to the general population, and this risk remains high for more than 20 years after the initial melanoma diagnosis [9].

In order to be protected from the harms of ultraviolet rays, raising awareness about sun protection in society, increasing the level of knowledge, and implementing correct practices in people's daily routine are necessary for both preventive medicine and the protection of CM patients from secondary skin tumors. In this study, the knowledge levels and habits of melanoma patients regarding sun protection were questioned through surveys and compared with the control group. Additionally, sun protection manner patterns in melanoma patients were evaluated before and after diagnosis. It is anticipated that the study results will shed light for clinicians to inform both healthy volunteers and melanoma patients and for preventive medicine.

## 2 Material‐Method

The study was designed as a case control study, aiming to detect possible differences in sunlight (the most consistently reported modifiable risk factor for CM) avoidance knowledge and behaviors between CM patients and the control group (consisting of people who have no history of CM in themselves or their first‐degree relatives) and the situations, if any, that these differences are associated with. In addition, it was planned to determine whether there was a change in sun protection behavior in CM patients after diagnosis and the factors that may be associated with it. Unlike the control group, CM patients were administered the sun avoidance/protection behaviors questionnaire twice, asking them to indicate their pre‐diagnosis and post‐diagnosis (current) behaviors separately. Beforehand, it was checked whether the patients remembered how they behaved before diagnosis. All patients who agreed to participate in the survey stated that they remembered the period before diagnosis.

The surveys were developed taking into consideration the Turkish Dermatology Association's sun protection patient information recommendations. The questions were prepared in two groups to question the level of sun protection knowledge and level of sun avoidance/protection behaviors. The study was approved by the Istanbul University Faculty of Medicine Deanery Clinical Research Ethics Committee (file number: 2022/570) and complied with the Declaration of Helsinki.

The CM patients who were followed up at Istanbul University Oncology Institute and agreed to participate in the study were contacted face‐to‐face or by phone to fill out the surveys. The control group consisted of patients and their relatives who applied to the hospital with any complaints and had no history of melanoma in themselves or in their first‐degree relatives. Written informed consent was obtained from patients who agreed to participate in the face‐to‐face survey, and verbal informed consent was obtained from those who participated in the telephone survey and the control group. Medical information of the patients was accessed through digital or physical records with the obtain of managers of the institute.

The sample size calculation was made according to the G*Power 3.1.9.4 program. Accordingly, it was necessary to determine 3 factors: an alpha error of 5% (Type I error), a beta error of 5% (Type II error; potency 95%), and an impact factor according to Cohen (0.5; medium impact) [10]. Based on the available data, a total sample size of 236 people was calculated, including 79 for the case group and 157 for the control group. By adding 10% prediction error, the targeted sample size was calculated as 260. Of the 110 CM patients whose surveys were completed, 86 were selected by random sampling. Of the 508 controls, 174 were selected based on age at diagnosis and gender of CM patients.

Statistical analysis of the results was calculated with the SPSS v 20.0 program. The data conformity to the normal distribution was tested with the Kolmogorov–Smirnov test. Parametric data obtained were expressed as mean ± standard deviation values. Analysis of categorical variables in both subgroups was evaluated using the chi‐square test. Pre‐ and post‐diagnosis sun protection behaviors of melanoma patients, as scored on the questionnaire, were compared using Wilcoxon tests due to the non‐normal distribution of the data. Since the condition of normal distribution was not met, comparisons of numerical variables in two or three independent groups were made using the Mann–Whitney *U* (MWU) test or Kruskal–Wallis(KWt) test, respectively. To investigate the association between risk factors and the outcome variable, we employed logistic regression analysis. We utilized multivariate logistic regression to determine the odds ratios (ORs) associated with each risk factor while adjusting for potential confounders. The model was constructed by including all relevant predictors, which were selected based on theoretical considerations and prior research. Adjusted ORs and 95% confidence intervals (CIs) were reported to quantify the strength and direction of associations. A *p*‐value below 0.05 was considered statistically significant.

## 3 Results

### 3.1 Demographic Data and Physical Characteristics of Patients and Participants

The Male:Female (M:F) ratio of 86 cm patients was 1.52, and the median age was 51 ± 15 years. In the control group of 174 participants, the M:F ratio was 1.38 and the median age was 49 ± 10 years. The CM patients and the control group were similar populations in terms of gender and age (*p* = 0.139, *p* = 0.589, respectively). Demographic data of the patient and control group are summarized in Table 1. While 24.2% of CM patients were university graduates, this rate was 52.3% in control patients. The education level of melanoma patients was statistically significantly lower than the control group (*p* < 0.001). The hair color of the CM patients was statistically significantly lighter than the control group (*p* < 0.001). Compared to the control group, the presence of freckles was statistically significantly higher in CM patients (*p* < 0.001). According to multivariate logistic regression analysis, males were found to have a higher risk of developing melanoma compared to females (OR: 2.44, 95% CI: 1.043–5.739, *p* = 0.040). Individuals with a university‐level education or higher had a lower risk of melanoma compared to those with only primary school education (OR: 0.27, 95% CI: 0.097–0.780, *p* = 0.015). Freckles were significantly associated with a higher risk of melanoma, with those having freckles being more likely to develop melanoma (OR: 4.331, 95% CI: 1.577–11.829, *p* = 0.004). Other factors such as eye color, hair color, and history of bullous sunburn did not show a significant effect on melanoma risk (Table 2).

**TABLE 1:** Demographic data of the cutaneous melanoma patients and control group.

Features	Cutaneous melanoma patients (*N* = 86)	Control *N* = 174	Chi‐square test (*p*)
Gender			
Male *n* (%)	52 (60.5)	101 (58.0)	0.139
Age, years (median) (min‐max)	51 ± 15 (10–84)	49 ± 10 (13–80)	0.08*
Education level			
Primary school, *n* (%)	38 (44.2)	40 (23.0)	**< 0.001**
High school, *n* (%)	27 (31.4)	43 (24.7)	
University and above, *n* (%)	21 (24.4)	91 (52.3)	
Hair color**			
Brown	22 (51.2)	90 (51.7)	**< 0.001** ***
Black	9 (20.9)	71 (40.8)	
Yellow	11 (25.6)	13 (7.5)	
Red***	1 (2.3)	0 (0.0)	
Eye color**			
Brown	22 (51.2)	107 (61.5)	0.258
Black	0 (0.0)	9 (5.2)	
Blue	4 (9.3)	9 (5.2)	
Green	5 (11.6)	15 (8.6)	
Hazel	12 (27.9)	34 (19.5)	
Freckling			
Present	23 (26.7)	25 (14.4)	**0.001**
Absent	63 (73.3)	149 (85.6)	
Bullous sunburn			
Never happened	58 (67.4)	134 (77.0)	0.106
Once	9 (10.5)	19 (10.9)	
More than once	19 (22.1)	21 (12.1)	

*Note:* Bold font *p*‐values indicates statistical significance.

* Analyzed by Student's *t*‐test.

** Missing values are excluded.

*** Red hair was excluded.

**TABLE 2:** Multivariate logistic regression analyses in the entire group.

Risk factors	Odds ratio	95% CI	*p*
Gender			
Female	1		
Male	2.44	1.043–5.739	**0.040**
Education level			
Primary school	1		
High school	1.16	0.445–3.059	0.754
University and higher	0.27	0.097–0.780	**0.015**
Eye color			
Brown	1		
Black	∞	N/A	0.999
Blue	1.23	0.266–5.755	0.785
Green	0.93	0.240–3.660	0.927
Hazel	1.72	0.690–4.290	0.244
Hair color			
Brown	1		
Black	0.613	0.238–1.579	0.311
Yellow	2.397	0.756–7.598	0.138
Red	0	N/A	1.0
Freckling			
Absent	1		
Present	4.331	1.577–11.829	**0.004**
Bullous sunburn			
Never happened	1		
Once	1.480	0.478–4.585	0.496
More than once	0.640	0.174–2.352	0.502

*Note:* Bold font *p*‐values indicates statistical significance. Odds ratio values of ∞ or 0 indicate issues with model separation or sparse data, and confidence intervals are not applicable (N/A).

Abbreviation: CI, confidence interval.

### 3.2 Sun Protection Knowledge Levels

Participants in the control group thought that they had statistically significantly higher knowledge of how to protect themselves from the sun compared to CM patients (*p* = 0.001). (Table 3, 1st question). It was determined that the most common source of information for melanoma patients who said they knew how to protect themselves from the sun was doctors (82.5%), while the most common source of information for the control group was media sources (49%) (*p* < 0.001). Compared to CM patients, participants in the control group had more accurate information on 5 questions about sunscreen application methods (applying at least 30 min before sun exposure, renewing after 2–4 h in sunny environments, renewing after the sea/pool, renewing after sports, amount of application). (*p* < 0.001 for each of the 5 questions). Similarly, the control group had statistically significantly higher knowledge of the necessity of using hats and glasses on sunny days (*p* = 0.002, *p* < 0.001, respectively). Participants in both groups had a high level of knowledge that they should avoid exposure to direct sunlight between 10.00 and 14.00. Comparison of sun protection knowledge levels of control and CM patients is summarized in Table 3.

**TABLE 3:** Comparison of sun protection knowledge levels of cutaneous melanoma patients and control group.

Questions		Cutaneous melanoma patients	Control	Chi‐square test (*p*)
Do you know how to protect yourself from the sun?	Yes, *N* (%)	58 (67.4)	148 (85.1)	**0.001**
	No, *N* (%)	28 (32.6)	26 (14.9)	
If yes, from whom did you get the recommendations?	Relatives/Friends, *N* (%)	3 (5.3)	40 (27.2)	**< 0.001**
	Media, *N* (%)	7 (12.3)	72 (49.0)	
	Clinicians, *N* (%)	47 (82.5)	35 (23.8)	
Do you know that you should not go out in the sun between 10:00 and 14:00?	Yes, *N* (%)	56 (96.6)	138 (93.2)	0.362
	No, *N* (%)	2 (3.4)	10 (6.8)	
Do you know that you have to apply sunscreen 30 min before going out in the sun?	Yes, *N* (%)	38 (65.5)	135 (91.2)	**< 0.001**
	No, *N* (%)	20 (34.5)	13 (8.8)	
Do you know that you have to reapply sunscreen every 2–4 h in sunny days?	Yes, *N* (%)	26 (44.8)	114 (77.0)	**< 0.001**
	No, *N* (%)	32 (55.2)	34 (23.0)	
Do you know that you need to reapply sunscreen after swimming in the sea or pool?	Yes, *N* (%)	35 (60.3)	138 (93.2)	**< 0.001**
	No, *N* (%)	23 (39.7)	10 (6.8)	
Do you know that you need to reapply sunscreen after sport activities?	Yes, *N* (%)	32 (55.2)	127 (85.8)	**< 0.001**
	No, *N* (%)	26 (44.8)	21 (14.2)	
Do you know how much sunscreen you should apply?	Yes, *N* (%)	24 (41.4)	118 (79.7)	**< 0.001**
	No, *N* (%)	34 (58.6)	30 (20.3)	
Do you know you have to put on a hat before going out in the sun?	Yes, *N* (%)	46 (79.3)	139 (93.9)	**0.002**
	No, *N* (%)	12 (20.7)	9 (6.1)	
Do you know you have to wear sunglasses before going out in the sun?	Yes, *N* (%)	36 (62.1)	139 (93.9)	**< 0.001**
	No, *N* (%)	22 (37.9)	9 (6.1)	
Do you know you have to wear tight‐woven clothes before going out in the sun?	Yes, *N* (%)	31 (53.4)	78 (52.7)	0.923
	No, *N* (%)	27 (46.6)	70 (47.3)	

*Note:* Bold font *p*‐values indicates statistical significance.

### 3.3 Sun Avoidance/Protection Behaviors (SAPB)

It was observed that the frequency of sun avoidance and use of sun protection cream (never/rarely/sometimes/most of the time/always) was statistically significantly higher in the control group (*p* < 0.001 for both). Eighty‐nine and a half % of CM patients stated that they did not use sunscreen at all. The rates of using hats and sunglasses were found to be statistically significantly higher in the control group (*p* < 0.001 for both). Comparison of SAPB of control and CM patients are summarized in Table 4.

**TABLE 4:** Comparison of the sun avoidance/protection behaviors of cutaneous melanoma patients (before diagnosis) and control group.

Questions		Cutaneous melanoma patients	Control	Chi‐square test (*p*)
How often do you avoid the sun?	Never, *N* (%)	39 (45.3)	15 (8.6)	**< 0.001**
	Rarely, *N* (%)	15 (17.4)	22 (12.6)	
	Sometimes, *N* (%)	10 (11.6)	64 (36.8)	
	Often, *N* (%)	16 (18.6)	52 (29.9)	
	Always, *N* (%)	6 (7.0)	21 (12.1)	
How often do you apply sunscreen before going out in the sun?	Never, *N* (%)	77 (89.5)	59 (33.9)	**< 0.001**
	Rarely, *N* (%)	5 (5.8)	49 (28.2)	
	Sometimes, *N* (%)	1 (1.2)	42 (24.1)	
	Often, *N* (%)	2 (2.3)	13 (7.5)	
	Always, *N* (%)	1 (1.2)	11 (6.3)	
What is the sun protection factor (SPF) of the sunscreen you apply?	2–12 SPF, *N* (%)	0 (0.0)	1 (1.1)	0.310
	12–30 SPF, *N* (%)	2 (22.2)	33 (36.7)	
	> 30 SPF, *N* (%)	7 (77.8)	56 (62.2)	
Would you reapply sunscreen?	Yes, *N* (%)	4 (44.4)	33 (28.7)	0.261
	No, *N* (%)	5 (55.6)	82 (71.3)	
If yes, how often do you apply sunscreen?	Once, *N* (%)	4 (4.7)	24 (13.8)	0.386
	Every 4 h, *N* (%)	0 (0.0)	11 (6.3)	
	Every 2 h, *N* (%)	0 (0.0)	1 (0.6)	
On which parts of your body do you apply sunscreen in your daily routine?	Only face, *N* (%)	5 (62.5)	74 (51.1)	0.421
	Face and extremities, *N* (%)	3 (37.5)	68 (47.8)	
Do you wear a hat before going out in the sun?	Yes, *N* (%)	23 (26.7)	139 (93.9)	**< 0.001**
	No, *N* (%)	63 (73.3)	9 (6.1)	
Do you wear clothes that cover your body before going out in the sun?	No, *N* (%)	44 (51.2)	70 (47.3)	0.568
	Yes, *N* (%)	42 (48.8)	78 (52.7)	
Do you wear sunglasses before going out in the sun?	Yes, *N* (%)	34 (39.5)	139 (93.9)	**< 0.001**
	No, *N* (%)	52 (60.5)	9 (6.1)	
How often do you sunbathe in summer?	Every day, *N* (%)	7 (8.1)	18 (10.3)	0.079
	Several times a week, *N* (%)	20 (23.3)	59 (33.9)	
	Several times a month, *N* (%)	22 (25.6)	49 (28.2)	
	Never, *N* (%)	37 (43.0)	48 (27.6)	
Have you ever used an indoor tanning bed?	Yes, *N* (%)	4 (4.7)	25 (14.4)	**0.019**
	No, *N* (%)	82 (95.3)	149 (85.6)	

*Note:* Bold font *p*‐values indicates statistical significance.

We scored the SAPB of CM patients as negative and positive to evaluate them before and after diagnosis. When we compared the SAPB of melanoma patients before and after diagnosis, we found that the diagnosis of melanoma had a significant positive effect (overall means of 11.00 and 39.63, respectively; Wilcoxon = −7.412, *p* < 0.001). (Table 5) When the positive change was examined in terms of independent subgroups (gender, age, and education), it was seen that there was no difference with gender (*p* = 0.25, MWU) and age (*p* = 0.14, KWt). Educational status (primary school/high school/university and above) was found to be statistically significantly associated with the change in behaviors after diagnosis (*p* = 0.005; KWt).

**TABLE 5:** Evaluation of sun avoidance/protection behaviors score before and after diagnosis in cutaneous melanoma patients (Wilcoxon Signed Ranks test).

		*N*	Mean rank	Sum of ranks	*p*
SAPB score after diagnosis–SAPB score before diagnosis	Negative ranks	3^a^	11.00	33.00	**< 0.001**
	Positive ranks	73^b^	39.63	2893.00	
	Ties	10^c^			
	Total	86			

*Note:* Bold font *p*‐values indicates statistical significance.

Abbreviation: SAPB, sun avoidance/protection behaviors.

^a^ SAPB score after diagnosis < SAPB score before diagnosis.

^b^ SAPB score after diagnosis > SAPB score before diagnosis.

^c^ SAPB score after diagnosis = SAPB score before diagnosis.

### 3.4 Nevus Screening Examination and Indoor Tanning Experience

The rate of melanoma patients and the control group having had a mole scan was equal. (2.3% for both) Experience of indoor tanning beds was significantly higher in the control group compared with CM patients. (*p* = 0.019).

## 4 Discussion

In our study, consistent with the literature, there was a 1.52‐fold male gender superiority in CM patients [11, 12]. In our study, the rate of high school and above education in melanoma patients was 55.4%, while this rate was 77% in the control group. In a study conducted by Wich et al. with minority melanoma patients living in the United States of America (USA), the rate of high school and above education was found to be between 63% and 72% according to different ethnic groups [13]. It appears that the education level of melanoma patients is lower in our country compared to the study reported in the USA. In addition, evaluating within the framework of our country, we observed that melanoma patients had a lower education profile compared to the control group of similar age and gender.

In the study by Vogel et al., no statistical difference was observed between melanoma patients and the control group in terms of educational status. In that survey study, it was determined that melanoma survivors exhibited healthier attitudes toward protecting themselves from UV rays than the control group [14]. In parallel, we can conclude that the low education level of the CM patients in the current study caused their sun protection knowledge to be less and their behavioral patterns to be more inappropriate. When melanoma survivors with similar education levels and the control group are compared in other studies, the fact that the melanoma survivor has a higher level of sun protection knowledge and a healthier behavior model may be due to medical oncologists or dermatologists giving detailed information to patients about sun protection and/or patients doing their own research after diagnosis. In our study, even being diagnosed with CM, which required mastering the principles of sun protection, could not overcome the factor of education level in terms of having a high level of sun protection knowledge and healthy behavioral patterns.

In another survey conducted with melanoma patients from our country, the rate of patients who said they had knowledge about sun protection was 93%, while in the current study, this rate was 67.4%, and the university graduation rate was found to be 53% and 24.4%, respectively [15]. The rates in these two studies conducted in our country reflect how effective the level of education is in having sun protection knowledge. The same study also reported that among melanoma patients, high education level was the only factor associated with awareness of malignant melanoma before diagnosis [15].

In a questionnaire based study conducted by Mueller et al. in which melanoma patients and people at high risk of melanoma participated, it was reported that sun protection creams with higher SPF (sun protection factor) were used as the education level increased [16]. On the other hand, the same study stated that as the education level increases, annual sun exposure also increases. Based on the study of Mueller et al., it can be concluded that the effect of high education level on the occurrence of CM is contradictory.

Another remarkable result of this study was that as the education level increased, the sun protection behaviors improved after diagnosis in melanoma patients. Nevertheless, this improvement was observed to be independent of gender and age. In addition to having a positive effect on the high level of knowledge and healthy behavioral patterns between the control group and CM patients, the high level of education is also a factor that has an impact on improving the behavioral patterns of CM patients after diagnosis. What makes the difference here may be that as the education level of post‐diagnosis CM patients increases, they will be able to better understand and more properly implement the recommendations of clinicians and to conduct more comprehensive and effective research on their own.

A study from the United States reported that people with high socioeconomic status may have an increased risk of CM formation due to their greater exposure to natural and artificial sunlight and regular holiday habits [17]. In our study, monthly salary was not questioned, so a definite comment cannot be made about socioeconomic status, but it is generally thought that as the level of education increases in our country, the socioeconomic level increases. From this perspective, in our study, high education level and the high socioeconomic level that may be associated with it seem to be a protective factor, not a risk factor, for CM occurrence.

Similar to our results, in the study of Vogel et al., freckling, one of the phenotypic risk factors of CM, was statistically significantly higher in melanoma patients [14]. While the rate of bullous sunburn in childhood was 56% in CM patients and participants in the melanoma‐risk group in the study by Mueller et al., we found this rate (in CM patients) to be 32.6% in our study [16].

## 5 Conclusion

As a salient result of our study, we detect that the education level in CM patients was statistically significantly lower than in the control group of similar age and gender. On the whole, this data may give an idea that the risk of CM increased in individuals with low education levels. In addition, in the control group, we considered parallel to the education level the level of sun protection knowledge was higher, and sun avoidance/protection behaviors were closer to what they should be. Moreover, the statistical significance between the improvement in sun protection behaviors after diagnosis and the higher level of education in CM patients indicates that the level of education may also be protective against secondary skin cancers in CM patients.

According to the results of our study, being male, having a low education level, and having freckles can be considered as possible risk factors for the development of cutaneous melanoma in our country. Even though the results of our study cannot be generalized because it is a single‐centred study, it may inspire oncologists, dermatologists, family physicians and public health professions regarding community‐based sun protection education studies. For instance, studies can be planned to investigate the rate of positive change in sun protection knowledge and habits in individuals who are shown standardized videos containing sun protection recommendations and explaining the application visually. If the effectiveness of the videos is proven, informational videos prepared by non‐profit organizations can be broadcast periodically in patient waiting rooms.

## Conflicts of Interest

The authors declare no conflicts of interest.

## Data Availability

The data (demographic data of patients and participants and also responds of questionnaires) used to support the findings of this study are available from the corresponding author upon request.
